# Endomyocardial, intralymphocyte, and whole blood concentrations of ciclosporin A in heart transplant recipients

**DOI:** 10.1186/2047-1440-2-5

**Published:** 2013-04-08

**Authors:** Ida Robertsen, Pål Falck, Arne K Andreassen, Nina K Næss, Niclas Lunder, Hege Christensen, Lars Gullestad, Anders Åsberg

**Affiliations:** 1 Department of Pharmaceutical Biosciences, School of Pharmacy, University of Oslo, P.O. Box 1068, Blindern, Oslo, 0316, Norway; 2 Department of Cardiology, Oslo University Hospital, Rikshospitalet, Oslo, 0027, Norway; 3 Center of Psychopharmacology, Diakonhjemmet Hospital, Oslo, 0319, Norway

**Keywords:** Ciclosporin A, Endomyocardial biopsies, Heart transplantation, Acute rejection, T-lymphocytes

## Abstract

**Background:**

In the early phases following heart transplantation a main challenge is to reduce the impact of acute rejections. Previous studies indicate that intracellular ciclosporin A (CsA) concentration may be a sensitive acute rejection marker in renal transplant recipients. The aims of this study were to evaluate the relationships between CsA concentrations at different target sites as potential therapeutic drug monitoring (TDM) tools in heart transplant recipients.

**Methods:**

Ten heart transplant recipients (8 men, 2 women) on CsA-based immunosuppression were enrolled in this prospective single-center pilot study. Blood samples were obtained once to twice weekly up to 12 weeks post-transplant. One of the routine biopsies was allocated to this study at each sampling time. Whole blood, intralymphocyte, and endomyocardial CsA concentrations were determined with validated HPLC-MS/MS-methods. Mann–Whitney *U* test was used when evaluating parameters between the two groups of patients. To correlate whole blood, intralymphocyte, and endomyocardial CsA concentrations linear regression analysis was used.

**Results:**

Three patients experienced mild rejections. In the study period, the mean (range) intralymphocyte CsA trough concentrations were 10.1 (1.5 to 39) and 8.1 (1.3 to 25) ng/10^6^ cells in the rejection and no-rejection group, respectively (*P*=0.21). Corresponding whole blood CsA concentrations were 316 (153 to 564) and 301 (152 to 513) ng/mL (*P*=0.33). There were no correlations between whole blood, intralymphocyte, or endomyocardial concentrations of CsA (*P* >0.11).

**Conclusions:**

The study did not support an association between decreasing intralymphocyte CsA concentrations and acute rejections. Further, there were no association between blood concentrations and concentrations at sites of action, potentially challenging TDM in these patients.

## Background

Heart transplantation is the final treatment option in end-stage heart failure and even though the procedure shows good results there is still room for improvement. In the early post-transplant phase a main challenge is to reduce the impact of acute rejections. The negative effects of the immunosuppressive therapy used to avoid acute rejection is however also a challenge in these patients. Hence, in the early phases following transplantation a combination of therapeutic drug monitoring (TDM) of immunosuppressive drugs and weekly endomyocardial biopsies are used to optimize the treatment for heart transplant recipients. A method with high specificity and accuracy to prevent graft rejection is an unmet clinical need.

Ciclosporin A (CsA) has been a cornerstone in the immunosuppressive therapy since its introduction in the mid 1980s. CsA is metabolized by the cytochrome P-450 3A (CYP3A) subfamily to >30 more or less pharmacologically active metabolites [1]. In addition, CsA is both a substrate and an inhibitor of the efflux transporter P-glycoprotein (P-gp) [2]. P-gp, coded by the *ABCB1* gene, is expressed in T-lymphocytes and transports CsA out of the cell [2-4]. A previous study has shown that polymorphism in the *ABCB1* gene may influence the intralymphocyte CsA concentration [5]. These pharmacokinetic properties are the basis for the substantial intra- and interindividual variation in CsA concentration. CsA is associated with a numerous of severe side effects, resulting in a narrow therapeutic range which makes the TDM of the drug extra demanding. The current routine TDM of CsA is performed by measuring whole blood concentrations, either in trough samples or lately also in C2 samples. However, since CsA exerts its immunosuppressive effect within T-lymphocytes [6], measurement of CsA within these cells may provide more relevant information regarding the immunosuppressive effect of CsA than whole blood concentrations. Several groups have shown data that support this hypothesis in transplant recipients [5,7-10]. We have recently shown that intracellular CsA concentration in T-lymphocytes decreased several days before an acute rejection was possible to diagnose in renal transplants by current standard clinical methods [7]. Intracellular CsA concentration monitoring therefore seems to have a potential as a semi-invasive method for prediction of acute rejection episodes. The purpose of the study was to evaluate the relationships between CsA concentrations at different target sites, that is whole blood, lymphocytes, and endomyocardial tissue, and to investigate CsA concentrations in isolated T-lymphocytes from heart transplant recipients in order to further examine intracellular monitoring as a potential TDM tool. In addition, the patients’ genotype of P-gp was determined to investigate if genetic polymorphism in the *ABCB1* gene could explain differences in the intralymphocyte concentration of CsA.

## Patients and methods

### Patients and study design

Ten heart transplant recipients (8 men and 2 women) with a mean age of 52 ± 12 years were enrolled in this single-center prospective pilot study. The patients were included 17 ± 6 days after transplantation and followed for a period of 70 ± 8 days. They all applied to standard post-transplant procedures at Oslo University Hospital, Rikshospitalet. All the patients were treated with C0-monitored CsA, mycophenolate mofetil (MMF), and steroids according to the center immunosuppressive protocol at that time. The CsA treatment was initiated with 10 mg/kg orally on the day of transplantation followed by C0 monitoring with target concentrations of 250 to 350 ng/mL after 1 month and further tapered to 60 to 120 mg/mL after 1 year of treatment. All patients received 1.5 g MMF twice daily from the day of transplantation, the doses was further adjusted according to side effects. The patients received 0.2 mg/kg/day oral prednisolone from the second postoperative day and were further tapered to 0.1 mg/kg/day within the following months. None of the patients were given induction therapy. Patients were not allowed to use concomitant drugs that could interact with CsA pharmacokinetics.

Study specific whole blood samples (EDTA vacutainer tubes) for CsA analyses and T-lymphocyte isolation were taken in association with routine blood samples for standard clinical follow-up; twice weekly during the first weeks and thereafter weekly samples for the rest of the investigation period. Whole blood samples and isolated T-lymphocytes were frozen and stored at −20°C until analysis. Routine monitoring of these patients include series of six endomyocardial biopsies at post-transplant week 1, 2, 5, 7, 10, and 12. One of the six biopsies taken at each time-point was allocated for CsA analysis in this study. The biopsy was wrapped in a piece of aluminum foil and stored at −20°C until analysis. In addition, EDTA whole blood was drawn once during the study for determination of the recipients *ABCB1* (*1199G>A*, *1236C>T*, *2677G>A*, *2677G>G*, and *3435C>T*) and *CYP3A5* (*3 (6986A>G, splicing defect)) genotypes. All acute rejections were verified with a biopsy and classified according to the International Society for Heart and Lung Transplantation (ISHLT) standardized cardiac biopsy grading [11,12].

The study was performed in accordance with the Declaration of Helsinki, local laws, and other regulations, and all patients signed a written informed consent before study start. The study was evaluated by the Regional Committee for Medical Research Ethics and approved by the Norwegian Medicines Agency. The study is registered on ClinicalTrials.gov (NCT00139009).

### T-lymphocyte isolation

T-lymphocytes were isolated from freshly drawn heparin whole blood using Prepacyte® (BioE, St Paul, MN, USA) [13]. An aliquot of 100 μM of verapamil was pre-added to the heparin vacutainers to inhibit P-gp from pumping CsA out of the cells [14]. Prepacyte® uses a negative selection process and facilitates the agglutination and precipitation of erythrocytes, B-lymphocytes, and mature myeloid cells like granulocytes, monocytes, and platelets, producing a supernatant of lymphocytes, highly enriched for T-cells. The excess of erythrocytes in the supernatant was removed by lysis using Vitalyse™. After centrifugation (400 g) and washing, the remaining supernatant contains >97% lymphocytes comprising 88% to 96% of the resultant cell population [15]. To relate the intracellular concentration to a physiological parameter, cell count using a *Bürker Chamber* was performed. The cells were isolated within 4 h post sampling. The isolating method starts with 7 mL of whole blood and produces a T-lymphocyte isolate pellet to which was finally added 1 mL methanol:ACN:water (1:1:3) for cell lysis and protein precipitation. The mixture was stored at −20°C until solid phase extraction and subsequent analysis of CsA concentrations.

### CsA and metabolite concentrations

Concentrations of CsA and six of its main metabolites were determined in whole blood, intracellularly in isolated T-lymphocytes, and in endomyocardial biopsies. The whole blood and intracellular CsA and metabolite concentrations were determined with a validated high-performance liquid chromatography-tandem mass spectrometry (HPLC-MS/MS) method previously described [16]. In brief, the analytes were extracted and purified by protein precipitation with methanol and centrifugation before the supernatants were subjected to solid phase extraction using Oasis hydrophilic-lipophilic balance cartridges. CsA and metabolites were separated chromatographically on a C8-colum before MS/MS detection. The intracellular concentration of CsA was related to the number of T-lymphocytes in the sample (ng/10^6^ cells).

The concentration of CsA and two metabolites, AM1 and AM9, were determined in endomyocardial biopsies by using a modification of the method described above [16]. After moistening the biopsy with 20 μL water for 5 min, the biopsy was weighed before homogenized in 150 μL deionized water with an automated tissue homogenizer; Precellys® 24 (Bertin Technologies, France), programmed to 2×50 s cycles with a 20-s pause. Fifty μL of the internal standard (0.5 μg/mL ciclosporin C (CsC) in methanol: acetonitrile (ACN): water (1:1:3)) was added to 100 μL homogenate and this mixture was protein precipitated with 100 μL ACN. Particulate matter was removed by centrifugation (30 min, 12,000 g, 4°C) and the supernatant was evaporated to dryness under a stream of nitrogen gas. The eluate was reconstituted in 50 μL of 65% mobile phase A consisting of ACN/20 mM ammonium formate buffer (NH_4_^+^COO^-^) pH 3.6 (20:80 v/v), and 35% mobile phase B, consisting of ACN/ NH_4_^+^COO^-^ (80:20 v/v), before injecting 20 μL on the LC-MS/MS system. The analytical system consisted of Aquity ultra performance liquid chromatography™ (UPLC) connected to a Micromass Quattro micro™ triple quadrupole mass spectrometry (MS) detector (Waters Corporation, USA) using electrospray ionization (ESI) interface. The detector was operated in a positive ion mode. Separation of the analytes was carried out on a reversed phase UPLC C18 column (100 × 2.1 mm, 1.7 μm) (Acquity UPLC BEH Shield C18, Waters, USA) and the column was heated to 70°C. The analytes were eluated using a stepwise gradient at the flow rate of 0.6 mL/min. The gradient program was as follows: 62% mobile phase A for 14 min followed by a gradually increase of mobile phase B to 100% for 7 min. One hundred per cent mobile phase B was held constant for 10 min and the system was finally re-equilibrated at start conditions for 5 min. Analysis run time per sample was 36 min. Calibration curves were produced from stock solutions of CsA, AM1, and AM9, which were mixed with the internal standard (CsC), evaporated to dryness under a stream of nitrogen gas and reconstituted in 65% mobile phase A and 35% mobile phase B. All standard curves comprised of at least eight concentration levels, including a blank sample (0.0 to 80 ng/mL). The regression coefficients (r^2^) of the linear standard curves were >0.998 and for both CsA and the metabolites the validation parameters for precision and accuracy (intra- and inter-run) were <9%.

### Genotyping

Genotyping was performed as previously described, using a polymerase chain reaction (PCR) - restriction fragment length polymorphism assay [17]. Restriction enzyme digestion generated DNA fragments that were separated by electrophorese on 3% agarose gels. All the patients were screened for relevant polymorphism in *CYP3A5* (*3 (6986A>G, splicing defect)) and *ABCB1* (*1199G>A*, *1236C>T*, *2677G>T*, *2677G>A*, *2677G>G*, and *3435C>T*). Dr D Katz (Abbott Laboratories, Abbot Park, IL (MDR1)) and Dr R van Schaik (Department of Clinical Chemistry Erasmus MC, The Netherlands (CYP3A5)) kindly supplied positive controls.

### Statistics and calculations

Mann–Whitney *U* test was used when evaluating parameters between the two groups of patients. To correlate whole blood, intralymphocyte, and endomyocardial CsA concentrations linear regression analysis was used. Statistical significant differences were considered for *P* values <0.05. All statistical analyses were performed using SPSS version 19. The renal function was estimated using the Modification of Diet in Renal Disease (MDRD) formula [18,19].

## Results

### Patients

All 10 heart transplant recipients completed this 3-month-long pilot study. Three patients experienced biopsy-proven acute rejection episodes during the study at an average of 58 ± 16 days after transplantation, and one of these patients experienced in total three rejection episodes during the study period. One of the patients in the no-rejection group developed renal failure during the study. Demographic data at inclusion are summarized in Table 1. No significant differences were observed between the rejecting and the no-rejection patients.

**Table 1:** Demographic data at time of inclusion

	**All**	**No-rejection group**	**Rejection group**	***P*value**
Gender (male/female)	8/2	2/5	3/0	-
Weight (kg)	76.7 ± 18.0	73.9 ± 19.5	83.3 ± 15.0	0.517
Age (years)	51.9 ± 11.9	51.0 ± 12.9	54.0 ± 11.5	0.833
CsA dose (mg/day)	330 ± 115	293 ± 116	417 ± 57.7	0.183
CsA C0 (ng/mL)	245 ± 59.3	239 ± 71.7	257 ± 10.4	0.383
Plasma creatinine (μmol/L)	131 ± 55	146 ± 59.8	96.5 ± 16.5	0.117
Creatinine clearance (mL/min)	58.0 ± 21.4	50.3 ± 18.9	77.6 ± 14.7	0.067
Serum urea (mmol/L)	10.5 ± 5.3	10.8 ± 6.0	9.8 ± 3.8	1.000
Hematocrit (%)	32.3 ± 4.2	32.3 ± 4.9	32.5 ± 0.7	0.500
Steroid dose (mg/day)	14.8 ± 3.8	13.6 ± 3.7	17.5 ± 2.5	0.137
Treated with MMF	10/10	7/7	3/3	-

Data are means ± SD. All variables were analyzed with a Mann–Whitney *U* test.

CsA, cyclosporine A; MMF, mycophenolate mofetil.

### Intracellular T-lymphocyte and whole blood concentrations of CsA

**Figure 1:**
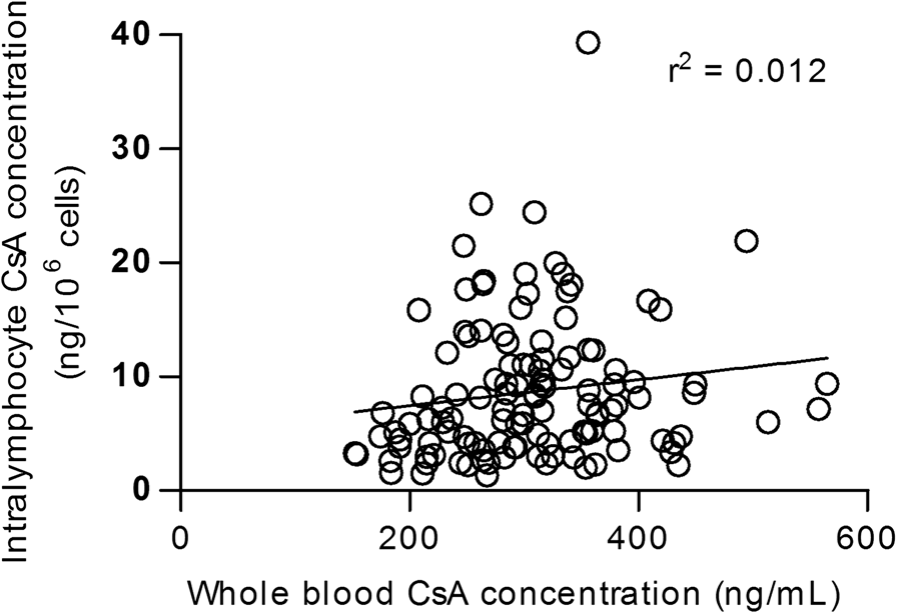
**Correlation between whole blood and intracellular CsA concentration in individual patients.** The figure shows all the whole blood and intracellular samples (*n* = 120) obtained during the study.

An average of 12.3 (range, 7 to 20) samples per patient was analyzed for both intracellular and whole blood concentration of CsA. In total, 139 whole blood samples and 121 intralymphocyte samples was analyzed during the study period. Both intracellular and whole blood concentrations of CsA showed large intra- and interindividual variations in both groups, and there were no correlation between whole blood and intracellular CsA concentration throughout the study (r^2^=0.012, *P*=0.11; Figure 1). In the study period, the mean (range) intracellular CsA trough concentrations were 10.1 (1.5 to 39) and 8.1 (1.3 to 25) ng/10^6^ cells in the rejection and no-rejection groups, respectively (*P*=0.21). The corresponding mean (range) whole blood CsA concentrations were 316 (153 to 564) and 301 (152 to 513) ng/mL, respectively (*P*=0.33).

**Figure 2:**
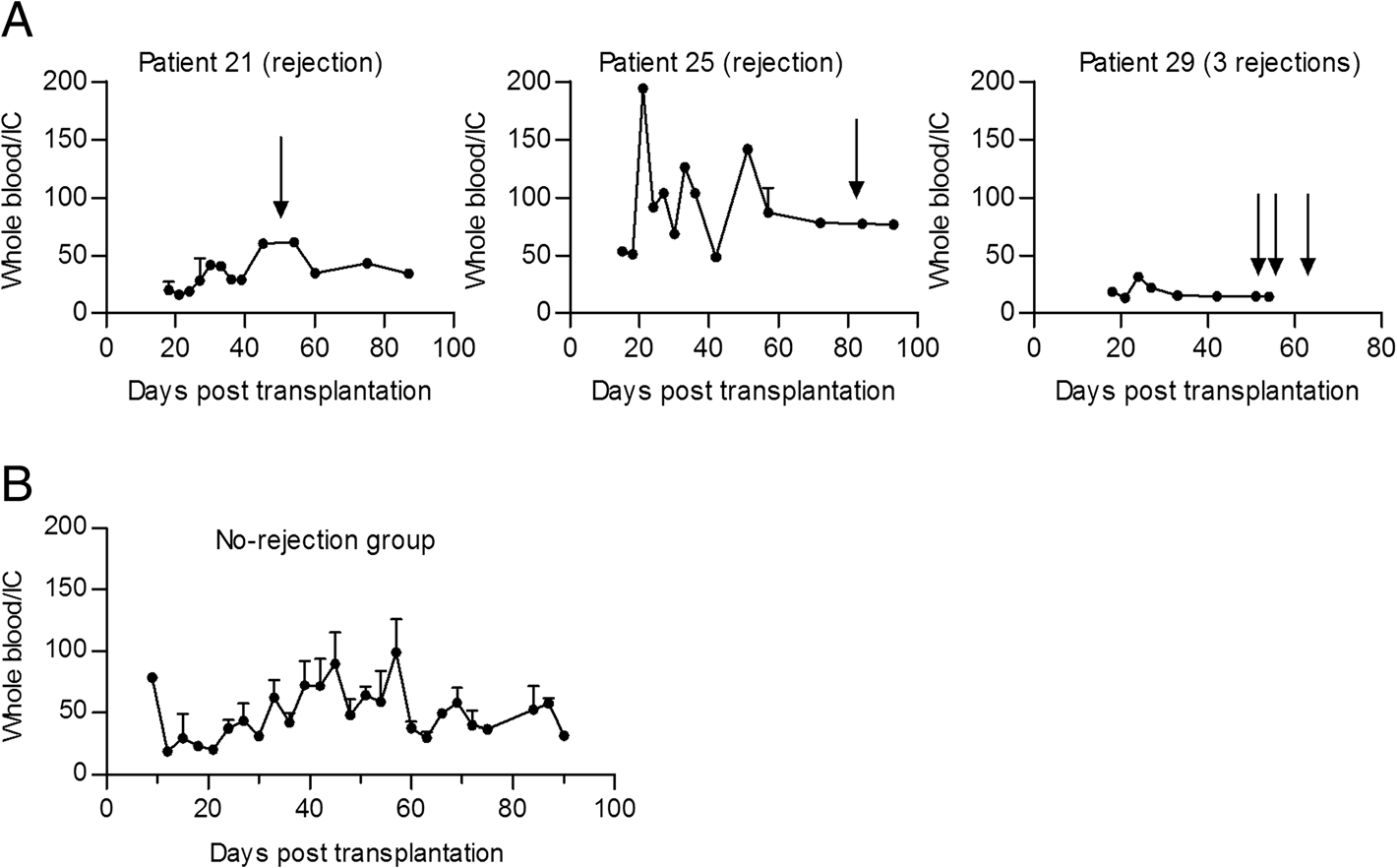
** The ratio (± SEM) of whole blood to intralymphocytic CsA trough concentration days after transplantation.** (**A**) The CsA whole blood/intracellular ratio (± SEM) for the three rejection patients. The arrows mark the point where the patients experienced an acute rejection episode. High levels of the ratio represent a drop in intracellular CsA concentration compared to whole blood concentration. (**B**) The mean whole blood/intracellular ratio (± SEM) for the patients with no rejection.

Figure 2 shows the individual ratio of whole blood to intralymphocytic CsA concentration for the three patients experiencing rejection and for the mean ratios for the no-rejection patients during the study period. One of the rejection patients (patient 21) showed an increase in the whole blood/intracellular ratio at time of rejection, due to a combination of declined intracellular concentration and a slight increase in the whole blood concentration. In the two other rejection patients (patients 25 and 29) no change was observed in the whole blood/intracellular ratio in conjunction to the rejection episode, but interestingly patient 25 showed substantially increased ratio on several occasions prior to the rejection episode as compared to the mean ratio for the no-rejection group (Figure 2A). In the no-rejection group the mean individual whole blood/intracellular ratio ranged from 33.6 to 86.4 with a corresponding standard deviation range of 17.8 to 46.7. The absolute average intracellular CsA concentration to the time of rejection was 10.4 (1.5 to 39) ng/10^6^ cells in the rejection group and the corresponding average CsA concentration to the mean time of rejection (day 58) was 8.2 (1.3 to 25) ng/10^6^ the no-rejection group (*P*=0.38). At the rejection day the absolute intracellular CsA concentration for the three rejecting patients were 9.4, 7.2, and 18. 4 ng/10^6^ cells.

### CsA metabolites, genotypes, and renal function

**Figure 3:**
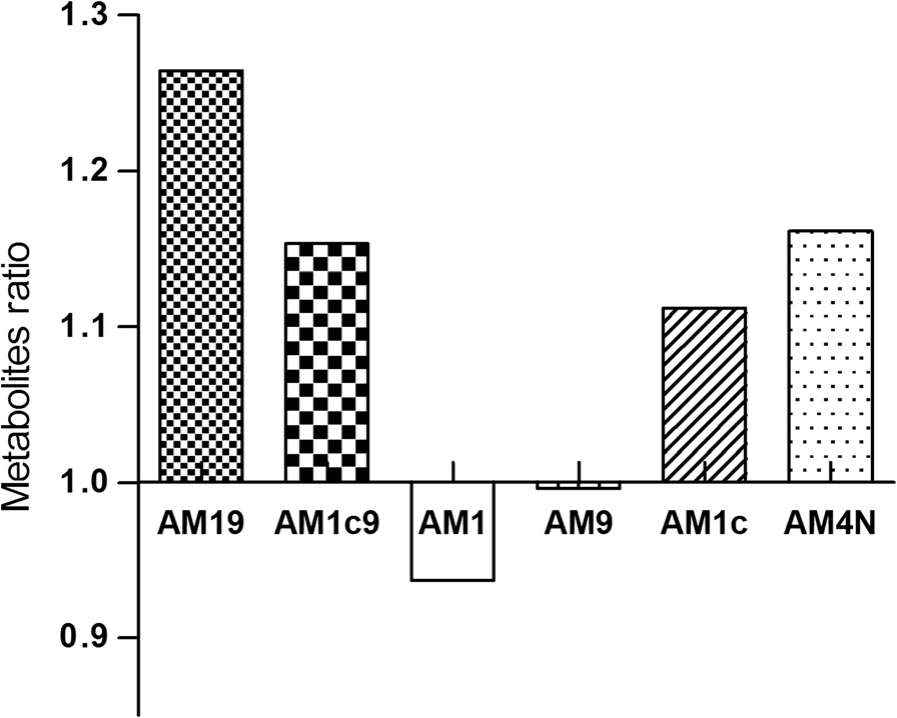
Ratio between the mean concentration of the metabolites AM19, AM1c9, AM1, AM9, AM1c, and AM4N in patients with CYP3A5*1/3 and in patients with CYP3A5*3/*3.

Genotyping results for both *ABCB1* (*1199G>A*, *1236C>T*, *2677G>T*, *2677G>A*, *2677G>G*, and *3435G>T*) and *CYP3A5* (*3) are presented in Table 2. Two out of three patients in the rejection group were homozygote *ABCB1* TTT carriers, but all patients were potential carriers of this reduced P-gp function haplotype. Three of the 10 patients expressed functional CYP3A5 enzymes (CYP3A5*1), one in the rejection group. It was observed that the patients expressing functional CYP3A5 enzymes tended to have higher concentrations of the metabolites AM19 (*P*=0.21), AM1c9 (*P*=0.57), AM1c (*P*=0.73), AM4N (*P*=0.27), similar concentration of AM9 (*P*=0.43), and a decreased concentration of AM1 (*P*=0.57) compared to the patients without functional CYP3A5 (Figure 3). We did not observe a significant difference in renal function between patients expressing functional CYP3A5 (eGFR of 51 ±23 mL/min) and patients not expressing functional CYP3A5 (eGFR of 66 ±19 mL/min, *P*=0.38). One of the three patients expressing functional CYP3A5 experienced renal failure during the study period.

**Table 2:** **Patient’s genotyping of *ABCB1 *and *CYP3A5***

**Patient**	***ABCB1***			***CYP3A5***
	**2677G>A/T**	**1236C>T**	**3435C>T**	*3
21	**T/T**	**T/T**	**T/T**	*3/*3
22	G/T	C/T	C/T	*1/*3
23	G/T	C/T	C/T	*3/*3
24	G/T	C/T	C/T	*3/*3
25	G/T	C/T	C/T	*3/*3
26	**T/T**	**T/T**	**T/T**	*3/*3
27	G/T	C/T	T/T	*1/*3
28	G/T	C/T	T/T	*3/*3
29	**T/T**	**T/T**	**T/T**	*1/*3
30	G/T	C/T	T/T	*3/*3

### Concentration of CsA and metabolites in endomyocardial biopsies

Nineteen biopsies, from seven out of the 10 patients, were obtained for the current study. Only one out of these seven patients was in the rejection group. In these patients an average of 2.7 (range, 1 to 6) biopsies per patient were analyzed for concentrations of CsA and two metabolites, AM1 and AM9. CsA concentration varied from 216 to 833 pg/mg heart tissue. No correlations were found between endomyocardial CsA concentrations and whole blood (r^2^=0.029, *P*=0.48) or intralymphocyte concentrations (r^2^=0.055, *P*=0.35). There was no obvious association between the endomyocardial concentration of CsA and rejection episodes.

## Discussion

The present pilot study does not support the hypothesis of decreased intracellular T-lymphocyte concentration of CsA prior to rejection episodes. The main finding, however, was that there were no correlations between CsA concentrations in whole blood, T-lymphocytes, or endomyocardial tissue.

Gustafsson and colleagues are, to our knowledge, the only group who previously has measured intralymphocyte CsA concentration in heart transplant recipients [10]. The study discovered a close association between whole blood CsA C2 concentrations and lymphocyte CsA AUC_0-12_ in MMF treated patients. This is contradictory to our findings where no correlation between CsA in whole blood and T-lymphocytes was found. A possible explanation to this discrepancy could be the fact that Gustafsson *et al.* performed measurement of whole blood CsA concentration in C2 samples and determined lymphocyte CsA AUC_0-12_, while in the present study CsA concentration were measured in C0 samples. C2 monitoring leads to an improvement in the clinical outcomes in heart transplant recipients [20,21] and measuring whole blood C2 concentrations could perhaps more precisely predict the CsA concentration and, in turn AUC, within lymphocytes. Nevertheless, our results are in agreement with previous studies reporting of no correlation between CsA concentration in whole blood and lymphocytes [22,23]. Although these studies were performed in different patient populations (renal transplant recipients and healthy volunteers), the findings demonstrate that whole blood CsA concentrations may not be a good predictor of the target site concentration of CsA. To the best of our knowledge, the present pilot study is the first to report of CsA concentration in endomyocardial tissue and to show the absence of correlation with both whole blood and intralymphocyte CsA concentrations in heart transplant recipients. In a recent study, Capron *et al.* evaluated the correlation of intrahepatic, peripheral mononuclear cells (PBMC) and blood concentrations of tacrolimus (Tac), another calcineurin inhibitor, in liver transplant recipients. In this study, no correlation was found between mean Tac blood concentration and PBMC or intrahepatic concentration of Tac. However, it was discovered that intrahepatic Tac concentration significantly correlated with Tac PBMC concentrations [24]. Capron *et al.* have earlier showed that hepatic tissue concentrations of Tac correlated with early acute rejection after liver transplantation, this in contrast to blood concentrations [25]. These findings also suggest that direct drug measurement at the target sites (lymphocytes and graft tissue) could be a better approach than measuring whole blood concentration to predict the efficacy of immunosuppressive drugs.

The present pilot study failed to show correlation between intracellular CsA concentration in T-lymphocytes and acute rejection episodes. Several other groups have however shown a possible correlation between low intracellular CsA concentration and rejection episodes in renal transplant recipients. A study conducted by Barbari *et al.* demonstrated that rejecting patients exhibited a low CsA lymphocyte content despite a higher or similar CsA blood concentration [8]. Similarly, we have shown that renal transplant recipients experiencing a rejection episode had a lower intracellular exposure of CsA several days before clinical diagnosis of acute rejection episodes [7]. The difference observed between renal and heart transplant recipients in this respect have no obvious explanation. However, as mentioned before C2 concentrations are known to correlate better with acute rejections compared to trough concentrations [20] and it was C2 concentrations that were used in our previous study [7]. Further, it cannot be ruled out that the renal transplant recipients experiencing an acute rejection episode had a stronger immune response compared to rejecting patients in the present study.

Since CsA is both a substrate and an inhibitor of P-gp, the patients’ genotype for this efflux pump was determined as it is expressed in T-lymphocytes. The *ABCB1* haplotype TTT (1236T, 2677T and 3435T) has previously been associated with impaired functional transport activity [26]. In the present study only three patients experienced an acute rejection episode. Two of the three rejection patients were homozygote *ABCB1* TTT haplotypes, but all patients included in the study were potential TTT haplotypes. This makes the interpretation of the data difficult, but if the hypothesis that acute rejection episode are associated with lower intracellular CsA concentrations should hold true, it would be expected that rejection patients have high transport activity of P-gp, contradictory to our findings [7,27].

Renal failure is a frequent and recognized complication following heart transplantation and CsA has been implicated as a potential risk factor [28-31]. Previous studies indicate that elevated blood and urine concentrations of the secondary metabolites AM19, AM1c, and AM1c9 may be associated with renal dysfunction in CsA treated patients [31-35], and that CYP3A5 expressers have higher formation of the secondary metabolites AM19 and AM1c9 [36]. Contrary, in renal transplant recipients on Tac-based immunosuppression, a protective role of CYP3A5 expression in the kidney has been observed [37]. By contrast to previous findings [31-35,38], the present study did not show any tendencies of a reduced renal function by an increased concentration of the secondary metabolites or functional CYP3A5 genotypes. This should however be carefully interpreted as the power is relatively low as outlined below.

### Study limitations

The main limitation of this pilot study is the low sample size and only three patients experienced acute rejection episodes. This clearly limits the conclusion that could be drawn. In addition, CsA concentrations were measured in trough samples and not in C2 samples. The intralymphocyte CsA concentration displayed a high intra- and interindividual variation, and this could partly be explained by the complex isolation procedure and the low level of automatization of the T-lymphocyte isolation method.

## Conclusions

The main finding of the present pilot study was that no correlation between CsA concentrations in whole blood, T-lymphocytes or endomyocardial tissue was present in heart transplant recipients. In addition, results from the present study do not support previous findings that CsA concentrations within T-lymphocytes decrease days before acute rejection episodes are diagnosed. The small sample size clearly limits the extent to which any definitive conclusion could be drawn. However, both findings are relevant with regards to TDM of CsA in this population and should be further investigated in properly powered clinical trials.

## Abbreviations

ACN: Acetonitrile; AUC: Area under the concentration versus time curve; C0: Concentration before dose (trough); C2: Concentration 2 hours after dose; CsA: Ciclosporin A; CsC: Ciclosporin C; CYP: Cytochrome P-450; HPLC-MS/MS: High performance liquid chromatography-tandem mass spectrometry; MDRD: Modification of diet in renal disease; MMF: Mycophenolate mofetil; PBMC: Peripheral blood mononuclear cells; PCR: Polymerase chain reaction; P-gp: P-glycoprotein; Tac: Tacrolimus; TDM: Therapeutic drug monitoring; UPLC: Ultra performance liquid chromatography

## Competing interests

The authors of this manuscript have no conflicts of interest to disclose.

## Authors’ contributions

PF, AKJ, LG, and AÅ designed the study and collected samples. AKJ and LG recruited patients. IR, PF, NKN, and NL analyzed data. IR and AÅ wrote the paper, whereas all authors have been involved in discussion of results and have contributed to, read, and approved the final manuscript.
